# Dark blood cine for improved Visualization of Intracavitary Structures (iVIS)

**DOI:** 10.1186/1532-429X-16-S1-P29

**Published:** 2014-01-16

**Authors:** Wolfgang G Rehwald, Elizabeth Jenista, David Wendell

**Affiliations:** 1 Siemens Healthcare, Chapel Hill, North Carolina, USA; 2 DCMRC, Duke University Medical Center, Durham, North Carolina, USA

## Background

In bright blood imaging, partial volume averaging of small moving intracavitary structures and surrounding bright blood often degrades the depiction of such morphology. Therefore, structures including papillary muscles, trabeculations, and intracardiac masses are better visualized with dark blood (DB) techniques. DB imaging also provides better image contrast for low signal structures since it avoids the dynamic range compression present in bright blood imaging. DB sequences are thus the standard imaging tool for assessing cardiac morphology, but they do not contain information about motion, for example of mobile intracardiac masses. We therefore developed a novel dark blood gradient echo cine sequence that simultaneously depicts morphology, motion of intracardiac structures, and cardiac contraction.

## Methods

**Figure 1:**
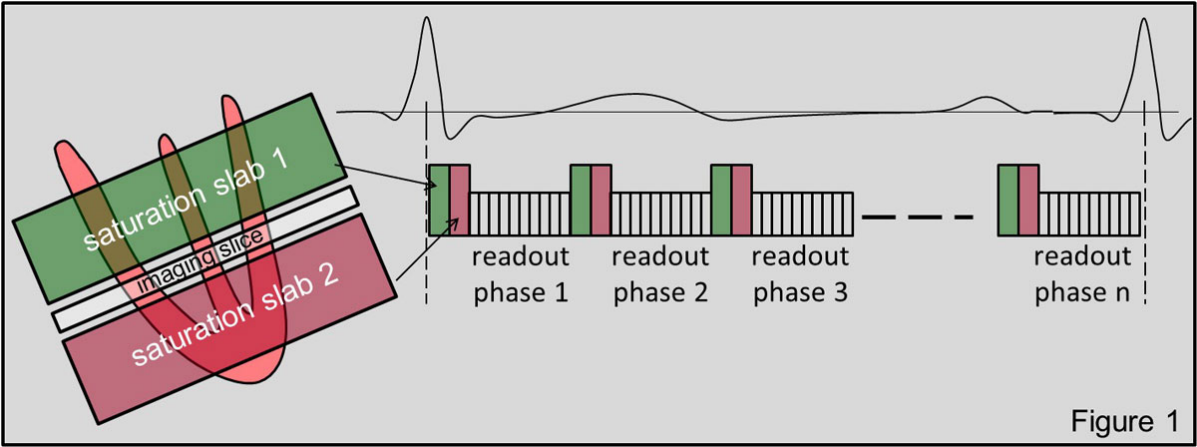
**location of imaging slice and parallel saturation slabs (left); sequence timing showing application the saturation slabs prior to segmented acquisition of each cardiac phase**. Only one RR interval is shown (right).

A prospectively gated spoiled gradient echo cine sequence (TE 2.28 ms, TR 5.0 ms, temporal resolution 35 ms, segments 7, matrix 256 × 140, fov 340 mm, slice thickness 3 mm) was modified to play a 75 mm saturation slab 5 mm above and 5 mm below the imaging slice before every cardiac phase (temporal resolution 48.15 ms), see Figure 1. On a Siemens MAGNETOM Verio 3 Tesla clinical MRI scanner, short-axis cine images were acquired in five volunteers with the conventional spoiled gradient echo cine sequence and with the new DB cine sequence. LV cavity and myocardium signal to noise (SNR) was measured in all cardiac phases of all patients and the average values of the conventional and the new DB cine images were compared.

## Results

**Figure 2:**
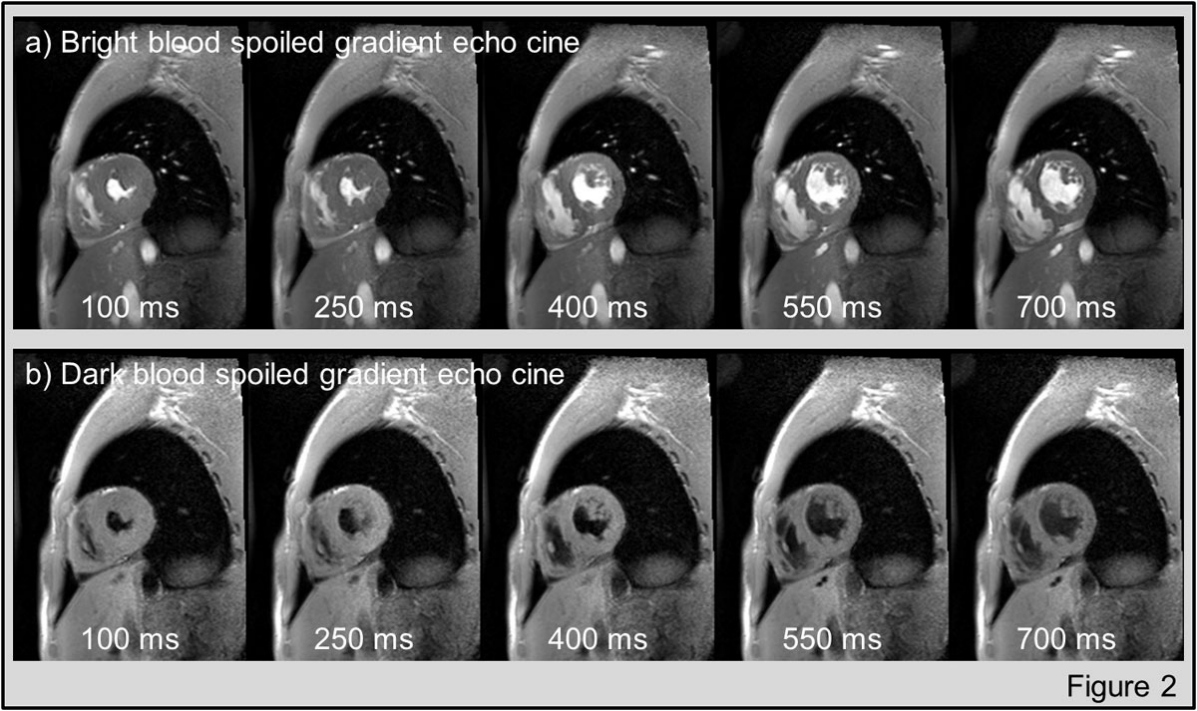
**panel a) shows representative cine frames (cardiac phases) acquired with the conventional bright blood cine sequence; panel b) shows the same frames acquired with the developed dark blood cine sequence**.

Figure 2 shows the same cine frames acquired with the conventional (panel a) and the developed DB cine sequence (panel b). Visual inspection revealed a strong reduction in blood signal. SNR measurements confirmed this finding. Average blood SNR was significantly reduced from 18.9 ± 4.9 to 3.4 ± 1.1 (mean ± stdev). Myocardial SNR was statistically identical in the conventional (13.3 ± 5.1) and dark blood (12.6 ± 2.9) images (p > 0.05).

## Conclusions

The presented DB cine technique reliably suppresses blood signal and thereby improves the depiction of intracardiac structures, masses, papillary muscles and trabeculations. The method is useful for simultaneously assessing cardiac function and morphology, for example in the setting of endocarditis or the presence of mobile masses.

## Funding

None.

